# Supplementation of a low-protein diet with tryptophan, threonine, and valine and its impact on growth performance, blood biochemical constituents, immune parameters, and carcass traits in broiler chickens

**DOI:** 10.14202/vetworld.2020.1234-1244

**Published:** 2020-06-30

**Authors:** Reham Abou-Elkhair, Hamada Ahmed, Sara Ketkat, Shaimaa Selim

**Affiliations:** 1 Department of Nutrition and Clinical Nutrition, Faculty of Veterinary Medicine, University of Sadat City, Egypt; 2 Department of Nutrition and Clinical Nutrition, Faculty of Veterinary Medicine, Damanhour University, Egypt; 3 Department of Animal Husbandry and Animal Wealth Development, Faculty of Veterinary Medicine, Alexandria University, Egypt; 4 Department of Nutrition and Clinical Nutrition, Faculty of Veterinary Medicine, Menoufia University, Shibin El-Kom, 32514, Egypt

**Keywords:** amino acids, broiler chickens, immune-related genes, meat composition, performance

## Abstract

**Aim:**

This study aimed to investigate the effects of protein reduction with supplementation of limiting amino acids (AA, tryptophan, threonine, and valine) on growth performance, blood biochemical, immunity parameters, and carcass traits in broiler chickens.

**Materials and Methods:**

Three hundred one-day-old broiler chicks were randomly allotted into three treatment groups, with five replicates per treatment and 20 broiler chickens per replicate. The three experimental diets were formulated with different dietary crude protein (CP) %, (control [CON] and CON with 1% [CP-1%] or 2% [CP-2%] less CP units) during the starter, grower, and finisher phases. The CP of the experimental diets were 22, 21, and 20% for the starting period (day 1-14); 20, 19, and 18% CP for the growing period (day 15-28); and 18, 17, and 16% CP for the finishing period (day 29-35) in CON, CP-1%, and CP-2%, respectively. The low-CP diets (CP-1% and CP-2%) were supplemented with combined AA, threonine+tryptophan+valine, to meet the respective levels of the CON diet.

**Results:**

The CP-2% group had greater (p<0.05; linear, p<0.05) final body weight and gain and better feed conversion ratio. The combined AA inclusion in the low-CP diet (CP-1% and CP-2%) increased (p<0.001; linear, p<0.001) carcass and breast meat yield as well as CP% of breast meat. The reduction of CP% with AA supplementation (CP-2%) decreased (p<0.05; linear, p<0.05) serum triglycerides, glutamate oxaloacetate transaminase, glutamate pyruvate transaminase, and alkaline phosphatase, while increased (p<0.01; linear, p<0.01) phagocytic activity and phagocytic index. The mRNA expression of splenic and cecal tonsil interleukin 4 and interferon gamma was upregulated (p<0.001; linear, p<0.001) in the low-CP diets supplemented with AA (CP-1% and CP-2%). Dietary supplementation with AA to low-protein diets improved (p<0.01; linear, p<0.01) the economic returns of broiler chickens.

**Conclusion:**

A reduction of dietary CP and increased synthetic AA such as threonine, valine, and tryptophan should be considered to improve performance, health, and immunity in broiler chickens.

## Introduction

Over the past few decades, one of the most important roles of nutritionists in the poultry industry is to reduce the feed cost while ensuring maximum efficiency of utilization and growth performance. Elevated ammonia concentrations in broiler houses have been reported to decrease growth performance, reduce immunity responsiveness, and respiratory problems in poultry [1,2]. Protein is considered one of the most expensive nutrients in commercial poultry rations. The use of synthetic amino acids (AA) is suggested to be an effective method for reducing feed costs and minimizing nitrogen excretion and environmental pollution [3-5].

Recently, it became possible to decrease dietary crude protein (CP) by 3% without a negative impact on growth performance in broiler chickens [6,7]. However, the use of low-protein diets at excessive levels of more than a 3% reduction in protein levels during the starter period was reported to have a negative impact on growth performance, carcass traits, intestinal morphology, and profitability [6-9]. The scenario becomes challenging when broiler chickens are fed low-protein diets supplemented with synthetic limiting AA. It is probable that the combination of these two strategies would result in better growth performance by providing a low-protein diet supplemented with commercially available synthetic limiting AA [5,10]. However, other studies have determined that this reduction can be disadvantageous, even with AA supplementation [11-13]. One possible reason for the reduced growth performance with these diets, in some studies, is that the supplementation with non-essential AA does not lead to performance recovery [13,14].

Few studies have been conducted to evaluate the effect of supplementation of low-protein diets with critical AA on growth performance and carcass traits, but little information is available on the outcomes of supplemented essential AA to low-CP diets on meat composition and the mRNA expression profiles of immunity-related genes (interleukin-4 [IL4] and interferon-gamma [IFN-γ]) in broiler chickens. There have been no studies regarding the regulatory effects of the combination of threonine, valine, and tryptophan supplementation to low CP diets. Emphasis is now being laid on investigating the impact of supplemented essential AA to low-CP diets at a molecular level. Moreover, it would be interesting to understand whether low CP diets supplemented with synthetic AA might be able to modulate the expression of cytokines (IL4 and IFN-γ) responsible for immunity in the gut of broiler chickens. Thus, our study was undertaken to investigate the effect of synthetic AA (tryptophan, threonine, and valine) supplementation to low-protein diets on growth performance, blood metabolites, carcass traits, meat composition, and the mRNA expression of immunity-related genes in broiler chickens that were fed low-protein diets. The results of this study will help in formulating diets for better growth performance, meat composition, and immune responsiveness of broiler chickens.

The hypothesis tested was that supplementation of a low-CP diet with synthetic tryptophan, threonine, and valine might improve growth performance, immunity, meat composition, and economic profitability of broiler chickens.

## Materials and Methods

### Ethical approval

All procedures in this study were approved by the animal care and use committee of the Faculty of Veterinary Medicine, Damanhour University, Egypt (Damanhour, Egypt).

### Study period and study location

The experiment was conducted at Faculty of Veterinary Medicine, Damanhour University, Egypt, during March to April 2018.

### Experimental design and bird husbandry

A total of 300 1-day-old Cobb 500 broiler chickens were randomly allocated to three experimental treatment groups, and each treatment group consisted of five replicates of 20 broiler chickens per pen (ten males and ten females). Broiler chickens were reared in raised floor pens from 1 to 35 day of age, at an optimum stocking density of 30 kg/m². Feed was provided in mash form, and the broiler chickens had free access to feed and water. The ambient temperature was adjusted at 32±2°C in the 1^st^ week; it was then reduced gradually to approximately 26±2°C and maintained at this temperature thereafter. Broiler chickens were reared under a continuous lighting program during the first 7 days (23 h light: 1 h darkness), and 16 h: 8 h L:D thereafter. The management of broiler chickens was consistent using the guidelines of the breeder standards and according to Cobb-Vantress [15]. The chicks were vaccinated against Newcastle disease, infectious bursal disease, and avian influenza according to the breeder standards. Mortality was checked daily in each pen.

### Experimental diets and feed analysis

The control (CON) diets were formulated to meet the requirements of broiler chickens as recommended by Cobb-Vantress [15], whereas the CP-1% and CP-2% diets were formulated with lower protein levels (less 1 protein unit, CP-1% and less 2 protein units, CP-2%) than the Cobb requirements. The three experimental diets were formulated to contain 22, 21, and 20% CP during the starter period (days 1-14); 20, 19, and 18% CP during the grower period (days 15-28); and 18, 17, and 16% CP during the finisher period (days 29-35) in CON, CP-1%, and CP-2%, respectively. The CP-1% and CP-2% diets were formulated to meet or exceed the recommendation for digestible AA according to Cobb-Vantress [15]. The percentages of studied AA in CON, CP-1% and CP-2% diets were as follow: Starter d-threonine (0.74, 0.78, and 0.91%), grower d-threonine (0.67, 0.69, and 0.71%), finisher d-threonine (0.55, 0.59, and 0.61%); starter d-tryptophan (0.23, 0.23, and 0.24%), grower d-tryptophan (0.20, 0.24, and 0.28%), finisher d-tryptophan (0.18, 0.18, and 0.17%); starter d-valine (0.78, 0.89, and 0.91%), grower d-valine (0.79, 0.83, and 0.85%), and finisher d-valine (0.76, 0.72, and 0.73%), respectively. The ingredients, chemical composition, and AA content of the basal diets are presented in Tables-1 and 2. Proximate analysis was conducted according to Association of Official Analytical Chemists (AOAC) [16] procedures. The AA content of the utilized ingredients was analyzed using near-infrared spectroscopy technology, multi-purpose analyzer, BRUKER (Hitachi, Inc., Tokyo, Japan) and is presented in Table-3.

**Table-1:** Composition of the experimental diets (as fed basis).

Items, g/kg	Starter (days 1 to 14)			Grower (days 15 to 28)			Finisher (days 29 to 35)		
		
	CON	CP-1%	CP-2%	CON	CP-1%	CP-2%	CON	CP-1%	CP-2%
Yellow corn	598	619	642	638	660	681	680	706	730
SBM, 46% CP	342	329	312	308	290	272	270	245	220
Corn gluten	20.0	10.0	0.0	10.0	5.0	0.0	0.0	0.0	0.0
Vegetable oil	3.0	3.0	3.0	10.0	9.0	8.0	17.0	14.0	12.0
Limestone	15.5	15.5	15.5	14.5	14.5	14.5	14	14.0	14.0
Monocalcium phosphate	10.0	10.0	10.0	9.0	9.0	9.0	8.0	8.0	8.0
L-Lysine^a^	1.7	2.2	3.0	1.3	2.0	2.8	1.1	1.8	3.05
DL-Methionine^b^	1.4	1.5	3.0	1.5	1.7	3.0	1.4	1.5	2.68
L-Threonine	0.0	0.79	1.58	0	0.50	0.99	0.0	0.35	0.7
L-Valine	0.0	0.04	0.076	0	0.11	0.22	0.0	0.025	0.05
Tryptophan	0.0	0.05	0.1	0	0.045	0.099	0.0	0.09	0.18
Common salt	2.0	2.0	2.0	2.5	2.5	2.5	3.0	3.0	3.0
Premix^c^	2.0	2.0	2.0	2.0	2.0	2.0	2.0	2.0	2.0
Na-bicarbonate	1.5	1.5	1.5	1.5	1.5	1.5	1.5	1.5	1.5
Choline	0. 90	0. 90	0. 90	0.50	0.50	0.50	0.50	0.50	0.50
Mycotoxin	0.50	0.50	0.50	0.50	0.50	0.50	0.50	0.50	0.50
Avizyme	0.20	0.20	0.20	0.20	0.20	0.20	0.20	0.20	0.20

The experimental diets were formulated to contain 22, 21, and 20 % CP for the starting period, 20, 19, and 18% CP for the growing period, and 18, 17, and 16% CP for the finishing period in CON, CP-1%, and CP-2%, respectively. The CP-1% and CP-2% groups were supplemented with threonine, valine, and tryptophan to meet or exceed the respective levels of the control diet.

a L-Lysine, lysine monohydrochloride by Feed Grade 99%.

b DL-Met, Met AMINO (DL-2-amino-4-(methyl-thio)-butane acid, DL-Met, a-amino-Y-methyl-oily acid) by Feed Grade 99%.

c Supplied per kilogram of diet: Vitamin A, 12,000 IU; Vitamin D3, 3,000 IU; Vitamin E, 40 mg; Vitamin K3, 3 mg; Vitamin B1, 2 mg; Vitamin B2, 6 mg; Vitamin B6, 5 mg; Vitamin B12, 0.02 mg; niacin, 45 mg; biotin, 0.075 mg; folic acid, 2 mg; pantothenic acid, 12 mg; Mn, 100 mg; Zn, 600 mg; Fe, 30 mg; Cu, 10 mg; I, 1 mg; Se, 0.2 mg; Co, 0.1 mg. ^d^ Each kg of Avizyme contains 4 g phytase, 40 g xylanase, 15 g amylase, 2 g pectinase, 8 g protease, 40 g beta glucanase, 40 g cellulase, 2.5 g methyl paraben, 1 g propyl paraben, and 847.5 g calcite

**Table-2:** The analyzed concentrations of nutrients of the experimental diets on an as fed basis^a^.

Items, %	Starter (days 1-14)			Grower (days 15-28)			Finisher (days 29-35)		
		
	CON	CP-1%	CP-2%	CON	CP-1%	CP-2%	CON	CP-1%	CP-2%
ME (kcal/kg)	3027	3030	3031	3105	3108	3110	3190	3195	3200
Analyzed CP	22.2	21.1	20.13	20.1	19.28	18.3	18	17.14	16.2
Calcium	1	1	0.99	0.94	0.93	0.93	0.84	0.89	0.87
Available phosphorus	0.45	0.45	0.45	0.42	0.43	0.42	0.40	0.40	0.40
Sodium	0.17	0.17	0.17	0.19	0.19	0.19	0.21	0.21	0.21
Digestible AA values									
dLysine	1.15	1.15	1.16	1.03	1.04	1.06	0.92	0.92	0.96
dMethionine	0.44	0.43	0.56	0.42	0.43	0.54	0.38	0.38	0.49
dMethionine+cystine	0.75	0.73	0.84	0.71	0.71	0.81	0.65	0.64	0.73
dThreonine	0.74	0.78	0.91	0.67	0.69	0.71	0.55	0.59	0.61
dTryptophan	0.23	0.23	0.24	0.20	0.24	0.28	0.18	0.18	0.17
dValine	0.78	0.89	0.91	0.79	0.83	0.85	0.76	0.72	0.73
dArginine	1.27	1.21	1.15	1.16	1.10	1.04	1.04	0.97	0.90
dIsoleucine	0.84	0.80	0.75	0.76	0.72	0.68	0.68	0.64	0.59

The experimental diets were formulated to contain 22, 21, and 20 % CP for the starting period, 20, 19, and 18% CP for the growing period, and 18, 17, and 16% CP for the finishing period in CON, CP-1%, and CP-2%, respectively. The CP-1% and CP-2% groups were supplemented with threonine, valine, and tryptophan to meet or exceed the respective levels of the control diet.

a Representative sample were analyzed in triplicate by NIR technology, MPA, BRUKER (Hitachi, Inc., Tokyo, Japan)

**Table-3:** Proximate analysis and amino acid content of the utilized ingredients^a^.

Items, %	Feedstuff		

	Yellow corn	SBM	Corn gluten
CP	7.5	46	62
ME	3350	2440	3500
EE	4.2	1.2	2
Moisture	12.2	12	10.9
DM	87.8	88	89.1
CF	2.2	3.2	0.8
Ash	1.2	5.5	1.5
Linoleic acid	2.18	0.4	3.17
Lysine	0.238	3.180	1.01
Methionine	0.172	0.720	1.43
Cystine	0.18	0.720	1.07
Methionine+Cystine	0.352	1.450	2.5
Threonine	0.295	1.900	2.02
Tryptophan	0.057	0.700	0.297
Valine	0.393	2.220	2.79
Isoleucine	0.278	2.120	2.43
Leucine	0.992	3.740	9.86
Phenylalanine	0.393	2.340	3.74
Histidine	0.246	1.280	1.24
Arginine	0.385	3.480	1.9
Serine	0.393	2.48	3.15
Glycine	0.319	2.05	1.6
Tyrosine	0.3	1.95	3.08
Ca	0.02	0.27	0.04
TP	0.27	0.29	0.46
Available P	0.09	0.24	0.138
K	0.34	1.98	0.16
Na	0.01	0.02	0.01
Mg	0.1	0.3	0.04
S	0.11	0.44	0.65
Cl	0.06	0.05	0.05

a Representative sample was analyzed in triplicate

### Data and sample collection

Broiler chickens and feed were weighed as a pen weekly during the experimental period for performance evaluation. Growth performance parameters, including body weight (BW), BW gain (BWG), and feed intake (FI), were measured, and feed conversion ratio (FCR) corrected for mortality was calculated. At the end of the experiment (35 days of age), 30 birds per treatment group (six per replicate) were randomly selected for determining carcass traits. Broiler chickens were eviscerated and dressed. Tissues from the liver, gizzard, heart, breast, spleen, thymus gland, and bursa of Fabricius were collected by removing the skin and connective tissue. In addition, breast muscle was isolated from the bone, and 30 uniform samples from each treatment were used to measure breast yield, moisture, dry matter (DM), CP, fat (EE), and ash % according to AOAC [16] procedures.

The blood samples (5 mL/broiler chicken) were collected from the jugular vein of six birds per replicate pen at 12, 24, and 35 days of age to obtain serum, which was directly aliquoted into 1-mL sterile vials and stored at −20°C until analysis. A hemagglutination inhibition (HI) test was performed to evaluate antibody titer according to the method described by Takatasy [17]. The serum samples at 35 days of age were also used for analyzing total serum protein, albumin, globulin, serum cholesterol, triglyceride, glucose, uric acid, creatinine, and glucose concentrations, glutamate oxaloacetate transaminase (GOT), glutamate pyruvate transaminase (GPT), and alkaline phosphatase (ALP) activity using commercially available kits (Biosystem S.A, Costa Brava, 30, Barcelona, Spain), according to manufacturer’s instructions. At 35 days of age, another set of blood samples (six samples per replicate) was aliquoted into 2-mL sterile vials containing anti-coagulant to measure phagocytic activity and phagocytic index, according to the procedure described by Kawahara *et al*. [18].

Gut immunity was evaluated by quantitative measurement of the mRNA expression of IL4 and IFN-γ in the cecal tonsils and spleen on day 35 of the experiment. Six birds from each replicate (30 per group) were slaughtered, and the intestines were collected carefully; the cecal tonsils and spleen were dissected and frozen at −80°C until analysis. The RNA extraction, preparation, and cycling conditions for real-time polymerase chain reaction (PCR) were performed as described previously by Bhanja *et al*. [19]. Primers and probes used in real-time PCR were described by Bhanja *et al*. [19] and are presented in Table-4. Amplification curves and cycle threshold (CT) values were assessed using Stratagene MX3005P software (Agilent Technologies, Inc., USA). To estimate the variation of gene expression on the mRNA level of the distinct samples, the CT of each sample was compared with that of the CON group according to the “DCt” method described by Yuan *et al*. [20]. The 28S rRNA was the reference housekeeping gene.

**Table-4:** Oligonucleotide sequence of growth- and immune-related gene primers [19].

Gene^a^	Sequence (5’®3’)	Annealing temperature (°C)	Accession Number	Product size (bp)	E, %^b^
IL-4	F-AATGACATCCAGGGAGAGGTTTC R-GCTAGTTGGTGGAAGAAGGTACG	55	JN639847	219	86
INF-γ	F-AGCTGACGGTGGACCTATTATTGT R-CGGCTTTGCGCTGGATTC	58	JN942588	260	86
28S rRNA	F-CAGGTGCAGATCTTGGTGGTAGTA R- GCTCCCGCTGGCTTCTCC	58	JN639848	273	98

a IL-4=Interleukin 4, IFN-γ=Interferon gamma; 28S rRNA=28 S ribosomal RNA,

b efficiency (E) was calculated from the slope of the standard curve using the equation: E=10 (−1/slope)

### Economic measurements

The economic analysis was performed initially considering FI and feeding costs per each live weight gain for all treatment diets during the experimental period. The analysis was calculated (FI during the respective period multiplied by the feeding cost per gram per each broiler chicken) according to the method described by Ebling *et al*. [21] and Ahmed *et al*. [22].

### Statistical analysis

The experiment was conducted using a completely randomized design with three treatments and five replicates, each containing 20 broiler chickens (ten males and ten females). The Kolmogorov–Smirnov test was used to test the normality of the experimental data before the statistical analysis. Experimental data were subjected to one-way ANOVA using IBM SPSS Statistics 22 statistical package (SPSS Inc., Chicago, IL, US) as a completely randomized design. The replicate pen was used as an experimental unit for analyzing the data. Significant differences among the experimental groups were determined using Tukey’s test at p<0.05. Linear and quadratic effects for the increment of AA in the diets were determined. The results are expressed as means ± SEM. Figures were prepared by GraphPad Prism 5 software (GraphPad Software, Inc., La Jolla, CA, US).

## Results

### Growth performance

The effects of supplementing combined synthetic AA profile (tryptophan, threonine, and valine) in a low-protein diet for broiler chickens during the experimental periods on BW, BWG, FI, and FCR are presented in Table-5. At 14, 28, and 35 days of age, broiler chickens fed diets with as much as a 2% CP reduction and received combined AA (CP-2% group) exhibited greater BW (CON vs. treatment, p<0.05; linear, p=0.001) compared to the CON and CP-1% groups. Broiler chickens in the CP-2% group at days 14 and 35 of age showed the lowest FI (CON vs. treatment, p<0.05; linear, p<0.01) compared to CON and CP-1%. Better FCR was recorded (CON vs. treatment, p<0.01; linear, p<0.01; quadratic, p=0.05) in the CP-2% group at days 14 of age compared to the CON and CP-1% groups. During the experimental period (from 1 to 35 days of age), the diets with a 2% CP reduction and combined AA supplementation (CP-2%) resulted in broiler chickens with greater final BWG (p<0.05; linear, p=0.01), lower FI (CON vs. treatment, p<0.01; linear, p=0.001; quadratic, p=0.05), and better FCR (p<0.01; linear, p<0.01; quadratic, p=0.01) than those that were receiving the CON and CP-1% diets.

**Table-5:** Effect of amino acid supplementation in a low-protein diet on body weight, body weight gain, feed intake, and feed conversion ratio during a 35-day production period.

Item	Treatments^a^			SEM	p-value		
	
	CON	CP-1%	CP-2%		CON versus treatment	Linear	Quadratic
Initial body weight, g	44.09	44.28	44.04	0.204	0.53	--	--
From 1 to 14 days							
Body weight (14 days), g	478.38^b^	487.09^b^	502.22^a^	4.08	0.003	0.001	0.39
Daily weight gain, g	31.02^b^	31.63^b^	32.73^a^	0.30	0.003	0.001	0.38
Daily feed intake, g	34.90^a^	35.19^a^	33.95^b^	0.19	0.002	0.003	0.004
Feed conversion ratio	1.13^a^	1.11^a^	1.04^b^	0.02	0.002	0.001	0.05
From 15 to 28 days							
Body weight (28 days), g	1341.44^b^	1358.73^ab^	1395.01^a^	14.53	0.03	0.01	0.48
Daily weight gain, g	61.64	62.26	63.77	0.95	0.15	0.06	0.60
Daily feed intake, g	103.50	103.45	101.00	1.19	0.13	0.08	0.29
Feed conversion ratio	1.68	1.66	1.58	0.04	0.08	0.04	0.36
From 29 to 35 days							
Body weight (35 days), g	1901.7^b^	1905.9^b^	1945.2^a^	11.95	0.02	0.01	0.14
Daily weight gain, g	80.03	78.17	78.59	1.94	0.63	0.49	0.52
Daily feed intake, g	139.71^a^	138.67^a^	134.95^b^	1.07	0.01	0.004	0.20
Feed conversion ratio	1.75	1.77	1.72	0.05	0.55	0.55	0.37
From 1 to 35 days							
Total body weight gain, g	1857.6^b^	1861.7^b^	1901.2^a^	11.867	0.02	0.01	0.14
Total feed intake, g	2915.7^a^	2911.7^a^	2834.0^b^	13.052	0.001	0.001	0.05
Feed conversion ratio	1.57^a^	1.56^a^	1.49^b^	0.012	0.001	0.001	0.01

^a,b^Means within rows with different letters are different at p<0.05; Tukey’s tests were applied to compare means; SEM=Standard error of the mean.

a The experimental diets were formulated to contain 22, 21, and 20 % CP for the starting period, 20, 19, and 18% CP for the growing period, and 18, 17, and 16% CP for the finishing period in CON, CP-1%, and CP-2%, respectively. The CP-1% and CP-2% groups were supplemented with threonine, valine, and tryptophan to meet or exceed the respective levels of the control diet

### Carcass traits

The effect of supplementing with combined synthetic AA in the low-protein diets for broiler chickens on carcass traits, relative weights of organs related to the immune system, and the proximate analysis of breast meat at the end of the experiment is presented in Table-6. No significant differences were observed in the gizzard and heart relative weights among the treatment groups. A greater dressing % (CON vs. treatment, p<0.001; linear, p<0.001) was found in broiler chickens receiving the CP-2% diet. The relative weights of the liver (p<0.05; linear, p=0.01) and breast meat (CON vs. treatment, p<0.001; linear, p<0.001) samples from broiler chickens receiving CP-1% and CP-2% diets revealed increased values compared with those fed the CON diet. Regarding immune system-related organs, the CP-1% and CP-2% diets resulted in broiler chickens with greater relative weights of the spleen (CON vs. treatment, p<0.001; linear, p<0.001; quadratic, p=0.07), thymus gland (p<0.001; linear, p<0.001, quadratic, p=0.01), and bursa of Fabricius (p<0.001; linear, p<0.001), within the normal range compared with CON chickens. The data are presented in Table-6 show a greater DM% of breast meat (CON vs. treatment, p<0.01; linear, p<0.01) in the CP-1% and CP-2% treatment groups compared with the CON group. The breast meat CP% of broiler chickens receiving the CP-2% diet had greater values (CON vs. treatment, p<0.01; linear, p<0.001) than those fed the CP-1% and CON diets. There were no significant differences in EE and ash% of breast meat between the treatment groups.

**Table-6:** Effect of amino acid supplementation in a low-protein diet on carcass traits and lymphoid organ weights (% of live body weight) of broiler chickens at the end of the experiment (35 days of age).

Item, %	Treatments^a^				p-value		
	
	CON	CP-1%	CP-2%	SEM	CON versus treatment	Linear	Quadratic
Dressing	74.52^c^	76.07^b^	77.21^a^	0.461	<0.001	<0.001	0.61
Liver	2.52^b^	2.64^ab^	2.66^a^	0.051	0.019	0.01	0.23
Gizzard	2.38	2.44	2.40	0.049	0.43	0.60	0.24
Heart	0.55	0.58	0.59	0.206	0.29	0.25	0.27
Breast	20.78^b^	21.57^a^	22.41^a^	0.417	<0.001	<0.001	0.38
Spleen	0.083^b^	0.091^a^	0.093^a^	0.002	<0.001	<0.001	0.07
Tdymus	0.372^b^	0.459^a^	0.479^a^	0.014	<0.001	<0.001	0.007
Bursa of Fabricius	0.115^b^	0.128^a^	0.136^a^	0.004	<0.001	<0.001	0.40
Breast meat proximate analysis							
Moisture	72.73^a^	71.64^b^	71.29^b^	0.409	0.004	0.001	0.30
DM	27.27^b^	28.34^a^	28.59^a^	0.397	0.005	0.002	0.24
CP	22.01^b^	22.33^b^	22.89^a^	0.208	0.001	<0.001	0.52
EE	2.80	2.78	2.77	0.031	0.62	0.33	0.98
Ash	2.46	3.22	2.93	0.456	0.25	0.31	0.19

^a,b^Means within rows with different letters are different at p<0.05; Tukey’s tests were applied to compare means; SEM=Standard error of the mean.

a The experimental diets were formulated to contain 22, 21, and 20% CP for the starting period, 20, 19, and 18% CP for the growing period, and 18, 17, and 16% CP for the finishing period in CON, CP-1%, and CP-2%, respectively. The CP-1% and CP-2% groups were supplemented with threonine, valine, and tryptophan to meet or exceed the respective levels of the control diet

### Blood biochemical constituents

Data regarding the blood biochemical constituents of broiler chickens fed the experimental diets are presented in Table-7. There were no significant differences in serum concentrations of albumin, albumin: Globulin, cholesterol, and glucose between the treatment groups. A significant increase (CON vs. treatment, p<0.01; linear, p<0.01) in serum total protein and globulin in broiler chickens of the CP-2% group compared to the CON and CP-1% groups. However, the CP-2% broiler chickens had the lowest concentration of serum total triglycerides (CON vs. treatment, p<0.01; linear, p<0.001), GOT (CON vs. treatment, p<0.01; linear, p<0.001), and creatinine (p=0.01; linear, p=0.01; quadratic, p=0.06) when compared with the CON and CP-1% broiler chickens. Conversely, broiler chickens receiving the CON diet had greater levels of serum uric acid, GPT, and ALP (p<0.05) than those fed the CP-1% and CP-2% diets. Significant increases (CON vs. treatment, p<0.01; linear, p<0.01) in the blood phagocytic index and phagocytic activity were found in the CP-2% group compared with the CON group.

**Table-7:** Effect of amino acid supplementation in a low-protein diet on blood biochemical constituents of broiler chickens at the end of the experiment (35 days of age).

Item^b^	Treatments^a^			SEM	p-value		
	
	CON	CP-1%	CP-2%		CON versus treatment	Linear	Quadratic
Total protein, g/dL	4.18^b^	4.50^b^	5.06^a^	0.124	<0.001	<0.001	0.29
Albumin, g/dL	3.06	3.16	3.40	0.153	0.12	0.05	0.61
Globulin, g/dL	1.12^b^	1.34^ab^	1.66^a^	0.120	0.003	0.001	0.64
A:G	2.79	2.41	2.08	0.278	0.08	0.03	0.92
Total cholesterol, mg/dL	207.0	205.8	206.7	1.81	0.61	0.34	0.85
Total triglycerides, mg/dL	200.8^a^	197.2^a^	192.4^b^	1.58	0.001	<0.001	0.67
Glucose, mg/dL	174.8	176.2	175.8	2.179	0.80	0.65	0.64
Uric acid, mg/dL	6.56^a^	5.52^b^	5.60^b^	0.311	0.01	0.009	0.06
Creatinine, mg/dL	0.520^a^	0.530^a^	0.468^b^	0.182	0.01	0.01	0.04
GOT, U/dL	77.00^a^	75.00^a^	71.80^b^	1.013	0.001	<0.001	0.51
GPT, U/dL	69.00^a^	67.80^ab^	65.70^b^	0.959	0.02	0.006	0.64
Alkaline phosphatase, U/L	12.34^a^	11.22^ab^	11.14^b^	0.424	0.03	0.02	0.18
Phagocytic index	3.57^b^	3.68^b^	4.84^a^	0.342	0.005	0.003	0.10
Phagocytic activity	61.80^b^	65.80^ab^	71.40^a^	2.372	0.006	0.002	0.70

^a,b^Means within rows with different letters are different at p<0.05; Tukey’s tests were applied to compare means,

SEM = Standard error of the mean.

a The experimental diets were formulated to contain 22, 21, and 20% CP for the starting period, 20, 19, and 18% CP for the growing period, and 18, 17, and 16% CP for the finishing period in CON, CP-1%, and CP-2%, respectively. The CP-1% and CP-2% groups were supplemented with threonine, valine, and tryptophan to meet or exceed the respective levels of the control diet.

b A:G=Albumin:globulin; GPT=Alanine aminotransferase; GOT=Aspartate aminotransferase

### Immune responses and immunity-related gene expression

**Figure-1:**
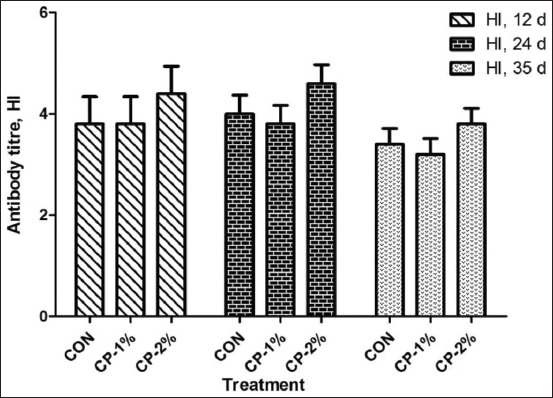
Effect of amino acid supplementation in a low-protein diet on antibody titer during a 35-day production period of broiler chickens. Data are presented as the mean±Standard error of the mean. Bars not sharing a common letter are different (p<0.05). The experimental diets were formulated to contain 22, 21, and 20 % crude protein (CP) for the starting period, 20, 19, and 18% CP for the growing period, and 18, 17, and 16% CP for the finishing period in control, CP-1%, and CP-2%, respectively. The CP-1% and CP-2% groups were supplemented with threonine, valine, and tryptophan to meet or exceed the respective levels of the control diet.

**Figure-2:**
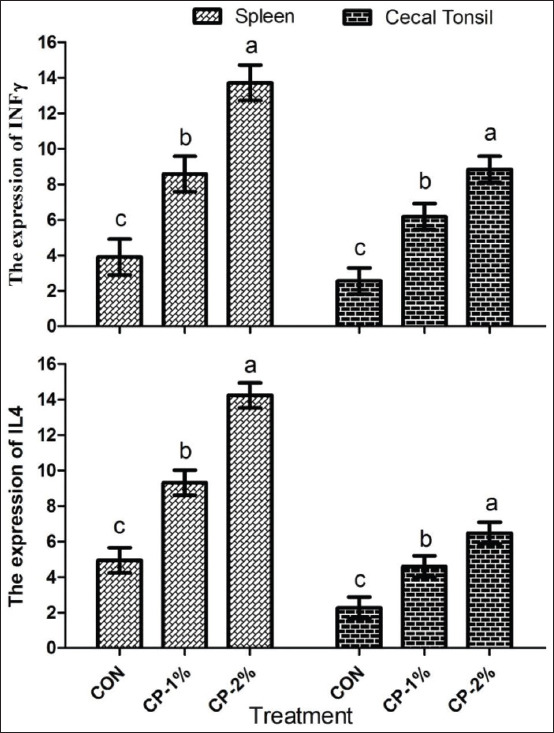
Effect of amino acid supplementation in a low-protein diet on the mRNA expression of cecal and splenic interleukin-4 (IL4) and interferon-gamma (INFγ) of broiler chickens at the end of the experiment (35 days of age). Data are presented as the mean±standard error of the mean. Bars not sharing a common letter are different (p<0.05). The experimental diets were formulated to contain 22, 21, and 20 % crude protein (CP) for the starting period, 20, 19, and 18% CP for the growing period, and 18, 17, and 16% CP for the finishing period in control, CP-1%, and CP-2%, respectively. The CP-1% and CP-2% groups were supplemented with threonine, valine, and tryptophan to meet or exceed the respective levels of the control diet.

Figure-1 shows serum antibody titers (HI) against Newcastle disease from broiler chickens receiving the experimental treatments during the experimental period. None of the treatments influenced (p>0.05) the serum antibody titers of the broiler chickens at 12, 24, or 35 days of age. The mRNA abundances of IL4 and INF-γ genes in the spleen and cecal tonsils are presented in Figure-2. The mRNA abundances of splenic interleukin 4 (IL4; CON vs. treatment, p<0.001; linear, p<0.001) and interferon gamma (INFγ; CON vs. treatment, p<0.001; linear, p<0.001) were upregulated in the CP-1% and CP-2% compared to the CON groups at the end of the experiment. In the cecal tonsils, greater mRNA expressions of IL4 and INFγ (CON vs. treatment, p<0.001; linear, p<0.001) were observed in the groups fed the CP-1% and CP-2% diet compared with those fed the CON diet. In addition, the lower mRNA expression of IL4 and INFγ in the spleen and cecal tonsils (p<0.001) was observed in the CON group compared with treatment groups.

### Economic profitability

The economic evaluation from the results obtained in Table-8 showed a significant difference between the CON group and the two treatment groups in total costs. The greatest total costs (p<0.001) were observed in the CON group (34.22 LE/bird). Moreover, the lowest total cost (linear, p<0.001) was found in the CP-2% group (33.05 LE/bird) followed by the CP-1% group (33.58 LE/bird). There is also a slight difference in the total return values among the CON and other treatment groups. The greatest total return (CON vs. treatment, p<0.05; linear, p=0.005) was obtained from the CP-2% group (49.63 LE/bird), and the lowest total return was (48.54 LE/bird) observed in the CON group. In addition, the net profit of the CP-% and CP-2% groups showed a significant improvement (CON vs. treatment, p<0.001; linear, p<0.001) as the highest net profit was obtained from the CP-2% group (16.58 LE/bird) followed by the net profit from the CP-1% group (15.07 LE/bird). The CON group showed the lowest net profit value (14.31 LE/bird).

**Table-8:** Effect of amino acid supplementation in a low-protein diet on the economic profitability of broiler chickens.

Items^b^	Treatments^a^				p-value		
	
	CON	CP-1%	CP-2%	SEM	CON versus treatment	Linear	Quadratic
Total cost	34.22^a^	33.58^b^	33.05^c^	0.052	<0.001	<0.001	0.22
Total return	48.54^b^	48.65^b^	49.63^a^	0.382	0.008	0.005	0.19
Net profit	14.31^b^	15.07^b^	16.58^a^	0.379	<0.001	<0.001	0.25

^a,b^Means within rows with different letters are different at p<0.05; Tukey’s tests were applied to compare means; SEM=Standard error of the mean.

a The experimental diets were formulated to contain 22, 21, and 20% CP for the starting period, 20, 19, and 18% CP for the growing period, and 18, 17, and 16% CP for the finishing period in CON, CP-1%, and CP-2%, respectively. The CP-1% and CP-2% groups were supplemented with threonine, valine, and tryptophan to meet or exceed the respective levels of the control diet.

b Total cost was calculated from the summation of total fixed cost and total variable cost. Total returns were the sum of litter sale and chicken sale. Chicken sale value = body weight at end of the experiment g× price. Net profit was the net income using the following equation: Net profit=Total return−Total cost

## Discussion

Considering the great interest in the use of low-CP diets in poultry nutrition, meeting the threonine, valine, and tryptophan requirements for optimal growth performance of broiler chickens is critical because these AA are considered essential AA in low-CP diets based on corn and soybean meal [3,5,10,14,23]. Waldroup *et al*. [14] also suggested that a balanced mixture of several synthetic AA may be more effective than supplementing a low-CP diet with a single essential AA.

### Growth performance

The linear positive effect for the CP-2% dietary treatment (2% less CP units+combined AA) on BW, BWG, and improved FCR of the broiler chickens throughout the experimental period was consistent with the results of earlier studies [14,23]. The previous studies reported that reducing dietary protein levels combined with essential AA supplementation had a positive impact on the growth performance of birds. This effect may be due to the greater availability of free crystalline AA than that in intact protein. Therefore, this composition of combined AA might play an important role in supporting optimal growth and various metabolic functions [14,24]. Moreover, this result may be due to dietary essential AA balance, efficient protein and AA utilization, and effective body capacity to meet the requirements of non-essential AA and an adequate pool of nitrogen to synthesize non-essential AA. In contrast, Namroud *et al*. [8] and Bregendahl *et al*. [12] found a reduction in the final BWs and BWG when CP in diets was reduced from 23% to 18% and fortified with essential AA. In the current study, the CP reduction was up to 2%, which is less than that reported by Namroud *et al*. [8] and Bregendahl *et al*. [12]. The growth retardation caused by deficiency of either individual or a combination of limiting AA such as lysine, methionine, valine, threonine, isoleucine, and tryptophan was well recognized in previous studies [3,10].

In our study, a 2% CP reduction combined with AA supplementation (CP-2%) resulted in low daily FI compared with the CON and CP-1% groups. This finding suggested that any excess AA (CP-2%) may be deaminated, producing more energy, which decreases FI as an animal defense to limit the adsorption and catabolism of excess AA [8]. In contrast, other studies reported no differences in FI of broiler chickens fed a low-protein diet supplemented with essential AA [25,26]. In line with the current findings of Opoola *et al*. [27] observed an increase in feed consumption in broiler chickens fed low CP diet supplemented with essential AA. These controversial reports may be attributed to several factors such as protein content of experimental diets, environmental factors, the phase of the experiment, and the digestibility of the supplemented AA. Inconsistencies between the results of previous studies and the current study may be attributed to the degree of dietary CP reduction, the age of broiler chickens, the AA used, and their digestibility, in addition to differences in the experimental design.

### Carcass traits and meat proximate analysis

The dietary treatments in our study had no effects on the relative weights of the gizzard and heart samples, but a reduction of CP% with AA supplementation linearly increased dressing %, breast meat yields, and relative weights of the liver samples. These findings were also reported in previous studies [10,24,27]. In contrast, Machado *et al*. [28] reported a reduction in breast meat yields with increasing digestible threonine in the diet. Furthermore, Ramarao *et al*. [29] demonstrated that carcass traits were not affected by the supplementation of threonine in a low-CP diet. The improvement in carcass traits in the present study may be due to increased essential AA (tryptophan, threonine, and valine), which plays important roles in the function of digestive enzymes, intestinal mucosa development and these AA are components of muscle protein [30].

It is noteworthy that immune organs (lymphoid organs) relative weights were linearly and quadratically increased (within the normal range) by AA supplementation in the low-protein diet. These results suggest greater activity and more antibody production, which resulted in an improvement of broiler immune responses [31,32]. These results agreed with the previous studies [33-36] who observed increased relative weights of the bursa of Fabricius, spleen, and thymus following dietary threonine and valine supplementation.

Regarding breast meat muscle proximate analysis, our results regarding the DM% were in line with previous studies [11,12]. Moreover, an increase in CP% in breast meat of the CP-2% broiler chickens was observed, which may be due to the increasing levels of essential AA, resulting in more protein deposition in the muscles. Conversely, Horniakova and Abas [37] reported no significant impact following AA supplementation in a low-protein diet on CP% of broiler chicken meat. Our findings revealed no effect for dietary treatments on fat and ash % of breast meat. However, Horniakova and Abas [37] observed lower fat % in muscles of the thigh and breast for birds fed a low-protein diet.

### Blood biochemical constituents

The analysis of blood biochemical constituents could help to evaluate the general health status of broiler chickens. The evaluation of serum levels of albumin, albumin: Globulin, cholesterol, and glucose showed no significant differences among treatments. Regarding serum glucose concentration, Opoola *et al*. [27] and Swennen *et al*. [38] revealed that carbohydrate metabolism was unaffected by AA supplementation in low-protein diets. Nevertheless, the dietary supplementation of combined AA (threonine, tryptophan, and valine) to a low CP diet linearly resulted in greater total protein and globulin values which indicate that the broiler chickens, in particular, the CP-2% group had the ability to maintain efficient homeostasis of AA and synthesis of protein in the tissue with an improvement in immune responses [7,39].

Lower serum total triglycerides in broiler chickens fed the CP-2% diet were observed. As FI of the low-CP diet group (CP-2%) was low, broiler chickens may have the ability to utilize body fat as an energy supply to compensate their feed efficiency [40], and thus resulted in low serum triglycerides. Creatine and uric acid serum levels are good indicators of protein and AA metabolism [41]. It is well-known that the rate of absorption of any synthetic AA is higher than that of intact protein [8]. The rapid flux of ingested supplementary free AA into the bloodstream may cause an imbalance in the plasma AA profile [42]. This could explain why the CON diet-fed broilers had higher serum levels of uric acid compared to the CP-1% and CP-2% groups. In this regard, Ospina-Rojas *et al*. [7] and Min *et al*. [39] reported the lowest plasma uric acid content when synthetic AA were added to the diet. On the contrary, Sigolo *et al*. [23] demonstrated that the reduction of dietary CP with threonine inclusion at its highest level (120 or 130 %) increased serum uric acid concentrations.

The plasma GOT, GPT, and ALP were used to evaluate the tissue damage in the liver and kidney [43]. GOT and GPT play key roles in mobilizing L-AA for gluconeogenesis and are considered a link between protein and carbohydrate metabolism [44]. GOT and GPT are greatly affected by AA (tryptophan, threonine, and valine) supplementation in a low CP diet. In our study, the lowest values for these enzymes were observed in the CP-2% treatment group compared with other treatments, suggesting hepatoprotective effects are associated with AA supplementation in a low CP-diet. This finding is supported by the findings of other studies [27,34,39]. Conversely, Chen *et al*. [32] demonstrated that higher dietary branched-chain AA greatly increase serum AST concentrations in chickens.

Enhanced phagocytic activity and phagocytic index in the AA supplemented groups, particularly CP-2%, suggested that critical AA supplementation in low-CP diets modulates immune functions [23,45] and that changes in the components of the immune system are sensitive to the diet [23]. Among AA, threonine and tryptophan have been proven to influence the immune system of broiler chickens [23,45]. The previous researchers reported that broilers fed a threonine supplemented diet had enhanced ND antibody titers compared with those fed a CON diet [23,29,36,39]. In line with the current findings, Fatemi and Toghyan [26] and Kaplan and Yildiz [35] demonstrated that there were no differences in antibody titers of broiler chickens fed diets supplemented with tryptophan and valine, respectively.

### Expression patterns of immune-related genes

The intestinal and splenic immune systems are crucial lines of defense and play important roles in animal protection from pathogenic microorganisms and toxins [46]. Cytokines, for example, IL4 and INFγ, are important protein mediators in humoral immunity, and the mRNA expression levels of cytokines were affected by proteins and AA levels in the diet [19,46]. In our study, the upregulation of gene expression of both IL4 and IFNg in cecal tonsils and spleens at the 35 days of age in the trial was linearly upregulated in the broiler chickens fed the CP-1% and CP-2% diets than in those of CON, which indicated higher humoral immune responses that could be attributed to high levels of threonine in immunoglobulins [19,46,47]. This finding corroborated an earlier report by Bhanja *et al*. [19] who found that *in ovo* injection of threonine increased the expression of humoral immune genes. This result also was in line with that of Han *et al*. [48], who observed that a deficiency of critical AA decreased the splenic and intestinal expression of cytokine genes. The current findings suggested better humoral and cellular immune response of broiler chickens fed a low-protein diet supplemented with three critical AA (threonine, valine, and tryptophan).

### Economic profitability

From the above results, we concluded that dietary supplementation with combined AA to a low-protein diet might improve the economic returns of broiler chickens. This result agreed those of with Ebling *et al*. [21] and Kidd *et al*. [49], who reported that the addition of supplements to broiler rations showed a slight increase in net revenues.

## Conclusion

In summary, our findings showed that a reduction of the dietary CP content combined with supplementation of three critical AA (threonine, valine, and tryptophan) did not negatively affect the growth performance of broiler chickens. A 2% reduction of dietary CP combined with AA supplementation decreased blood serum levels of uric acid, creatinine, GOT, GPT, ALP, and total cholesterol. Moreover, the inclusion of combined AA in a low-protein diet raised eviscerated carcass weight, and consequently carcass, breast, and meat yields. Enhanced phagocytic activity and phagocytic index were also noted in the treatment group receiving a 2% CP reduction and combined AA supplementation. Upregulation of the immune-related genes was also observed in the AA supplementation to a low CP diet. Overall, the current findings provided support for a reduction of dietary CP up to 2% during the starting, growing, and finishing periods, with 100% supplementation of AA (threonine, valine, and tryptophan) achieving better performance, health, and immunity of broiler chickens.

## Authors’ Contributions

HA, RA and SS conceived and conducted the experiment. RA and SS drafted the manuscript. RA, SS, HA and SK conducted data interpretation and edited the manuscript. SK conducted the economic data calculation. SS performed the statistical analysis, prepared tables and figures. All authors read and approved the final manuscript.
